# Linking Physical Activity Motivation Regulation to Health Behavior Participation Among Korean Adults: The Mediating Role of Self-Regulation Strategies

**DOI:** 10.3390/healthcare14121765

**Published:** 2026-06-18

**Authors:** Guifang Liu, Ryu Seok, Sung-Un Park, Deok-Jin Jang, Wi-Young So

**Affiliations:** 1 Department of Physical Education, Zhengzhou Normal University, Zhengzhou 450044, China; liuguifang@zznu.edu.cn; 2 Department of Taekwondo, Kyung Hee University, Yongin 17104, Republic of Korea; koku9@khu.ac.kr; 3 Department of Sports Science, Hwasung Medi-Science University, Hwaseong 18274, Republic of Korea; psu@hsmu.ac.kr; 4 Department of Sports Medicine, Shinhan University, Uijeongbu 11644, Republic of Korea; 5 Department of Sports Medicine, College of Humanities, Korea National University of Transportation, Chungju 27469, Republic of Korea

**Keywords:** autonomous motivation, controlled regulation, health behavior engagement, physical activity, self-determination theory, self-regulation strategies

## Abstract

**Objectives/Background**: Regular physical activity is a key health behavior associated with physical and mental well-being. However, sustaining physical activity remains challenging among adults, and the psychological mechanisms that support continued engagement require further clarification. Grounded in Self-Determination Theory, this study examined the roles of autonomous motivation and controlled regulation in health behavior engagement, focusing on the mediating role of physical activity self-regulation strategies. **Methods**: A cross-sectional study was conducted with 468 Korean adults. Autonomous motivation and controlled regulation were assessed using the Behavioral Regulation in Exercise Questionnaire-3, and physical activity self-regulation strategies were measured using the Physical Activity Self-Regulation Scale. Health behavior engagement was assessed using a single-item measure based on the stages of change for exercise. Structural equation modeling with bootstrapping was used to examine direct and indirect relationships among the study variables. **Results**: Both autonomous motivation (β = 0.649, *p* < 0.001) and controlled regulation (β = 0.153, *p* < 0.001) were positively associated positive with self-regulation strategies. Self-regulation strategies were positively associated with health behavior engagement (β = 0.336, *p* < 0.001). Autonomous motivation showed both a significant direct effect (β = 0.131, *p* = 0.018) and a significant indirect effects through self-regulation strategies (bias-corrected 95% confidence interval [CI] [0.206, 0.420]) on health behavior engagement, indicating partial mediation. In contrast, controlled regulation showed no significant direct effect (β = −0.062, *p* = 0.144) but had a significant indirect effect through self-regulation strategies (bias-corrected 95% CI [0.043, 0.131]). **Conclusions**: Autonomous motivation appears to be a stronger predictor of health behavior engagement than controlled regulation, both directly and indirectly through self-regulation strategies. These findings highlight the importance of motivational quality and suggest that self-regulation strategies are a key mechanism through which motivation is translated into physical activity engagement among adults. Interventions should therefore combine autonomy-supportive approaches alongside the development of practical self-regulatory skills to promote sustained physical activity.

## 1. Introduction

Exercise participation is a key health behavior that is associated not only with overall health maintenance and weight management but also with mental health outcomes, including anxiety and depression [1,2,3,4]. Despite these benefits, many adults struggle to maintain regular exercise participation over time [5,6,7]. Because exercise adherence cannot be fully explained by environmental conditions, health limitations, or awareness of its benefits, understanding the psychological mechanisms that support sustained engagement remains important [7,8]. These observations highlight the need to identify the psychological and behavioral mechanisms underlying engagement in health behaviors among adults.

Self-Determination Theory (SDT) provides a useful framework for examining exercise behaviors. SDT distinguishes between autonomous motivation, in which behavior is self-endorsed and personally valued, and controlled regulation, in which behavior is driven by external or internal pressures [9,10,11,12]. Previous studies have demonstrated that autonomous motivation is consistently associated with physical activity participation, whereas evidence for controlled regulation is less consistent [12,13,14]. These findings suggest that the quality of that motivation may play an important role in understanding health behavior engagement.

Motivational quality alone may not fully explain engagement in health behaviors. Research has shown that motivation does not always translate directly into action, highlighting the importance of self-regulatory processes that support behavior enactment and maintenance [8]. Physical activity self-regulation strategies, such as goal setting, self-monitoring, and time management, may therefore represent an important mechanism linking motivation to health behavior engagement [8,15,16]. Autonomous motivation is generally associated with greater use of these strategies and, consequently, higher levels of physical activity participation [12,13,16].

Although autonomous motivation, controlled regulation, and self-regulation strategies have each been linked to health behavior outcomes, relatively few studies have examined these relationships within a single structural model. In particular, relatively few studies have investigated whether the higher-order dimensions of autonomous motivation and controlled regulation are associated with health behavior engagement through integrated physical activity self-regulation strategies [17,18,19]. Furthermore, when health behavior engagement is conceptualized not merely as exercise volume but as the level of participation based on the stages of change for exercise, limited research has examined how self-regulation strategies mediate the relationships between autonomous motivation, controlled regulation, and such engagement outcomes [20,21,22]. This gap is important because stage-based engagement reflects adults’ readiness, enactment, and maintenance of exercise behavior, rather than only the amount of physical activity performed.

Therefore, this study examined the relationships among exercise-related motivational regulation, physical activity self-regulation strategies, and health behavior engagement within the framework of SDT. By testing both the direct and indirect pathways, this study aims to clarify the mechanisms underlying sustained engagement in health behavior among Korean adults.

## 2. Theoretical Background

SDT distinguishes between autonomous and controlled forms of motivation based on the degree to which behavior is self-endorsed [9,10,11,12].

In the exercise context, autonomous motivation reflects participation driven by personal value and choice, whereas controlled regulation reflects participation motivated by external pressures or obligation [11,12,13]. Previous research has consistently linked autonomous motivation to greater physical activity participation, while findings for controlled regulation have been less consistent [12,13].

Motivation alone may not be sufficient to explain health behavior engagement. Self-regulation strategies are important because they help individuals translate motivation into sustained action [7,8,15]. In the context of physical activity, self-regulation strategies include goal setting, self-monitoring, time management, social support seeking, reinforcement, and relapse prevention all of which have been associated with exercise participation and maintenance [23,24,25].

Accordingly, the present study conceptualizes physical activity self-regulation strategies as an integrated self-regulatory resource that supports the planning, enactment, and maintenance of exercise behavior.

Health behavior is conceptualized as a participation construct reflecting readiness for and engagement in exercise behavior [20,22,26]. Consistent with prior research, engagement is assessed using the stages of change framework, which classifies individuals according to their level of exercise participation and maintenance [27,28]. Consistent with prior research, engagement is assessed using the stages of change framework, which classifies individuals according to their level of exercise participation and maintenance [27,28].

Previous findings suggest that self-regulation strategies may represent an important mechanism linking motivation to health behavior engagement. From an SDT perspective, autonomous motivation may facilitate the use of self-regulation strategies—such as planning, self-monitoring, and coping with barriers—because exercise is experienced as self-endorsed and personally meaningful [12,13,16]. These findings suggest that self-regulation strategies may mediate the relationship between exercise-related motivational regulation and health behavior engagement.

Although motivation and self-regulation have each been associated with physical activity outcomes, relatively few studies have examined their relationships within a single structural model that simultaneously considers autonomous motivation, controlled regulation, self-regulation strategies, and health behavior engagement [19,20,25]. Therefore, the present study examines whether physical activity self-regulation strategies mediate the relationships between exercise-related motivational regulation and health behavior engagement.

**H1.**  *Autonomous motivation and controlled regulation will each have significant effects on physical activity self-regulation strategies.*

**H2.**  *Autonomous motivation and controlled regulation will each have significant effects on health behavior engagement.*

**H3.**  *Physical activity self-regulation strategies will mediate the effects of autonomous motivation and controlled regulation on health behavior engagement.*

## 3. Materials and Methods

### 3.1. Participants

A total of 468 adults were included in the final analysis. Of these, 227 participants were male (48.5%) and 241 were female (51.5%). Detailed background characteristics of the participants are presented in Table 1. Sample size adequacy was evaluated using an a priori power analysis conducted in G*Power (G*power 3.1.9.7, Heinrich-Heine-University, Düsseldorf, Germany). For a linear multiple regression model with three predictors, assuming a medium effect size (f^2^ = 0.15), an alpha level of 0.05, and a target power of 0.95, the minimum required sample size was estimated to be 119. Given that the present study included 468 participants, the sample size was deemed sufficient.

### 3.2. Data Collection

Data were collected from Korean adults aged 19 years or older through an online survey conducted between July and December 2025 using a non-probability convenience sampling method. The survey was administered via Google Forms, and participants were recruited through public distribution across various online channels and through an adult online panel maintained by a professional survey company. Because this recruitment procedure relied on online access and voluntary participation, it was not intended to produce a population-representative sample. Accordingly, the possibility of selection bias related to age distribution, digital accessibility, and exercise-related interest should be considered when interpreting the findings. A total of 513 responses were initially obtained. After excluding cases with substantial missing data, repetitive identical responses indicative of careless answering, and cases that did not meet the study eligibility criteria, 468 responses were retained for the final analysis. Prior to participation, all respondents were provided with information regarding the purpose and procedures of the study, the voluntary nature of participation, and the assurance of anonymity, and they proceeded with the survey only after providing informed consent. This study was conducted after approval from the Institutional Review Board (IRB) of Hwasung Medi-Science University (approval number: HSMUIRB-2025-03; approval date was 1 March 2025), and all procedures were carried out in accordance with the ethical principles of the Declaration of Helsinki for research involving human participants.

### 3.3. Measures

#### 3.3.1. Exercise-Related Motivational Regulation (BREQ-3)

Exercise-related motivational regulation was assessed using the BREQ-3, a measure grounded in SDT and designed to assess motivational regulation in exercise behavior. In the present study, the version proposed by Cid et al. [29] was adapted and refined for the purposes of this study. The BREQ-3 typically consists of six subdimensions: amotivation, external regulation, introjected regulation, identified regulation, integrated regulation, and intrinsic motivation. However, because the present study focused on the structural relationships between active forms of exercise-related motivational regulation and levels of health behavior engagement, amotivation was excluded from the analysis. In line with the conceptual distinctions proposed by SDT, external regulation and introjected regulation were classified as controlled regulation, whereas identified regulation, integrated regulation, and intrinsic motivation were classified as autonomous motivation in the analyses [10,11,30].

#### 3.3.2. Physical Activity Self-Regulation Strategies (PASR-12)

Physical activity self-regulation strategies were measured using the PASR-12 developed by Umstattd et al. [23]. The PASR-12 is a multidimensional scale that encompasses six substrategies: goal setting, self-monitoring, time management, eliciting social support, reinforcement, and relapse prevention. It was developed as a brief and valid measure of self-regulation strategies in the context of physical activity. In the present study, the mean scores of the six substrategies were used as observed indicators to construct a single higher-order latent variable representing physical activity self-regulation strategies [24].

#### 3.3.3. Health Behavior Engagement (Single-Item Measure)

Health behavior engagement was assessed using a single-item measure based on the stages of change for exercise [27]. This item classifies respondents into five stages—precontemplation, contemplation, preparation, action, and maintenance—and may be understood as an ordinal indicator reflecting both readiness for and enactment of health behavior. The specific wording of the item was as follows: “Which of the following best describes your current exercise participation?” Respondents selected one of five options: 1 = “I do not currently exercise and do not intend to start exercising within the next six months”; 2 = “I do not currently exercise but intend to start exercising within the next six months”; 3 = “I currently exercise, but not regularly”; 4 = “I currently exercise regularly, but have been doing so for less than six months”; and 5 = “I currently exercise regularly and have been doing so for six months or longer.” In the context of physical activity, the single-item self-classification approach has demonstrated agreement comparable to that of parallel assessment methods [28], and recent efforts to redevelop exercise behavior measures have retained stage of change as a core component [26]. In the present study, responses were coded from 1 to 5, with higher scores indicating higher levels of health behavior engagement. Based on methodological discussions suggesting that five-category ordinal data may, under certain conditions, be treated as approximately continuous in structural models, this variable was included as the dependent variable [31,32]. Accordingly, the scores were interpreted as indicating the relative ordering of health behavior engagement rather than equal interval differences between categories.

### 3.4. Validity and Reliability

To evaluate the validity and reliability of the instruments used in this study, confirmatory factor analysis (CFA) and reliability analyses were conducted. The results are presented in Table 2. For the exercise-related motivational regulation scale, the measurement model demonstrated acceptable overall fit to the data: χ^2^ = 224.485, df = 80, minimum discrepancy divided by degrees of freedom (CMIN/df) = 2.806, incremental fit index (IFI) = 0.965, Tucker–Lewis index (TLI) = 0.954, comparative fit index (CFI) = 0.965, and root mean square error of approximation (RMSEA) = 0.062. Standardized factor loadings ranged from 0.518 to 0.935. Composite reliability (CR) values ranged from 0.799 to 0.937, average variance extracted (AVE) values ranged from 0.581 to 0.832, and Cronbach’s α coefficients ranged from 0.748 to 0.935, indicating acceptable convergent validity and internal consistency. For the physical activity self-regulation strategies scale, the measurement model also demonstrated satisfactory fit: χ^2^ = 109.180, df = 39, CMIN/df = 2.799, IFI = 0.979, TLI = 0.964, CFI = 0.979, and RMSEA = 0.062. Standardized factor loadings ranged from 0.747 to 0.959. Composite reliability (CR) values ranged from 0.805 to 0.883, AVE values ranged from 0.674 to 0.792, and Cronbach’s α coefficients ranged from 0.804 to 0.880, indicating satisfactory reliability and validity. Overall, these results suggest that the subfactor structures of the scales demonstrated adequate validity and reliability, supporting the use of theoretically grounded higher-order parsimonious constructs in subsequent structural model analyses.

### 3.5. Statistical Analysis

Data were analyzed using IBM SPSS Statistics 23.0 and IBM SPSS Amos 23.0 (IBM Corp., Armonk, NY, USA). Statistical significance was set at *p* < 0.05. First, for the BREQ-3 and PASR-12, reverse-coded items were processed in accordance with the scoring guidelines, and mean scores were calculated for each subfactor. Health behavior engagement was measured using a single-item indicator based on the stages of change for exercise algorithm proposed by Marcus et al. [27]. Respondents were classified into five stages—precontemplation, contemplation, preparation, action, and maintenance—based on their exercise behavior, duration of participation, and intention to initiate exercise. The variable was recoded such that higher scores indicated higher levels of engagement.

This single-item self-classification approach has been used in physical activity research and has demonstrated comparable patterns to parallel assessment methods [28]. In addition, recent efforts to redevelop exercise behavior measures have continued to treat stages of change as a core measurement component [26]. Accordingly, in the present study, the variable was treated as an ordinal outcome reflecting the level of health behavior engagement and was included as the dependent variable in the structural model, based on methodological support for approximating five-category ordinal data as continuous in such analyses [31,32].

Confirmatory factor analyses for the BREQ-3 and PASR-12 were conducted using maximum likelihood estimation to examine the factor structure and reliability of each scale. Model fit in the confirmatory factor analyses was evaluated using χ^2^ together with the CFI, TLI, and RMSEA. Model adequacy was interpreted based on multiple fit indices rather than a single cutoff criterion [33,34,35,36].

Descriptive statistics, including means, standard deviations, skewness, kurtosis, and Pearson correlation coefficients, were calculated to examine distributional characteristics and the bivariate relationships among variables. Multicollinearity was also assessed. Univariate distributional characteristics were evaluated using skewness and kurtosis; following Curran et al. [37], absolute skewness values below 2.00 and absolute kurtosis values below 7.00 were considered to indicate no serious departure from univariate normality. Pearson correlation coefficients were interpreted in terms of statistical significance and magnitude, with absolute values of 0.10–0.39, 0.40–0.69, and ≥0.70 indicating weak, moderate, and strong associations, respectively [38,39].

Because the study focused on structural relationships and indirect effects rather than item-level variation, a parsimonious modeling approach was adopted. Specifically, mean scores of subfactors or theoretically homogeneous composite indicators were used instead of individual items. This approach reduced model complexity while preserving essential measurement information, and was justified based on construct dimensionality, theoretical coherence, and evidence for measurement validity [40,41,42,43].

Finally, the simplified structural model was analyzed using maximum likelihood estimation to estimate direct and indirect effects. As the model became saturated following simplification, global model fit indices were not interpreted at the structural level. Instead, results were interpreted primarily in terms of path coefficients and indirect effects. The significance of indirect effects was evaluated using nonparametric bootstrapping with bias-corrected 95% confidence intervals; and significance was determined based on whether the confidence interval included zero [44,45]. The composition of each construct included in the final structural model is presented in Table 3. In this study, latent constructs were operationalized using mean scores of subfactors to reduce complexity and facilitate a clearer examination of the structural relationships.

## 4. Results

### 4.1. Descriptive Statistics and Correlations Among the Study Variables

The results of the descriptive statistics and correlation analyses for the study variables are presented in Table 4. As indicated in the table, external regulation was significantly and negatively correlated with identified regulation, integrated regulation, intrinsic motivation, most self-regulation strategies, and health behavior engagement. In contrast, variables reflecting autonomous motivation—namely identified regulation, integrated regulation, and intrinsic motivation—demonstrated generally significant positive correlations with the subfactors of self-regulation strategies and with health behavior engagement. Furthermore, health behavior engagement was significantly negatively correlated with external regulation, whereas it was significantly positively correlated with introjected regulation, identified regulation, integrated regulation, intrinsic motivation, and all subfactors of self-regulation strategies. Regarding normality, the skewness values of the study variables ranged from −1.110 to 1.093, and the kurtosis values ranged from −0.884 to 0.937, indicating that the assumption of univariate normality was not substantially violated. Although health behavior engagement showed the largest negative skewness (skewness = −1.110), this value remained within the prespecified criterion for univariate distributional characteristics. Thus, the skewness was not considered severe enough to challenge the assumptions of the analysis. However, because health behavior engagement was measured as a five-category ordinal stage-based indicator, the results should be interpreted as reflecting ordered levels of engagement rather than equal-interval differences in actual physical activity volume. Overall, this pattern of correlations suggests that the subfactors retained conceptually distinct characteristics while being related to one another in theoretically consistent directions. These findings support the application of higher-order parsimonious constructs in the subsequent structural model analysis. This apparent difference should be interpreted in light of the level of analysis. External regulation was examined as a single subfactor in Table 4, whereas controlled regulation in Table 5 was modeled as a higher-order construct including both external and introjected regulation.

### 4.2. Structural Equation Modeling Analysis

The path coefficients for the simplified final structural model are presented in Table 5. As indicated in Table 5, both autonomous motivation and controlled regulation had significant positive effects on self-regulation strategies. Notably, the effect of autonomous motivation (β = 0.649, *p* < 0.001) was substantially greater than that of controlled regulation (β = 0.153, *p* < 0.001), indicating that autonomous motivation had stronger explanatory power in the development of self-regulation strategies. In addition, self-regulation strategies had a significant positive effect on health behavior engagement (β = 0.336, *p* < 0.001), suggesting that self-regulation strategies may function as a key mechanism linking motivation and behavior. With respect to the direct paths to health behavior engagement, only autonomous motivation demonstrated a significant effect (β = 0.131, *p* = 0.018), whereas the direct effect of controlled regulation was not significant (β = −0.062, *p* = 0.144). Overall, these findings indicate that autonomous motivation contributes to health behavior engagement both directly and indirectly through self-regulation strategies. By contrast, controlled regulation did not show a significant direct effect on health behavior engagement and may therefore operate primarily through indirect pathways involving self-regulation strategies. The squared multiple correlations indicated that autonomous motivation and controlled regulation explained 44.5% of the variance in self-regulation strategies (R^2^ = 0.445). In addition, autonomous motivation, controlled regulation, and self-regulation strategies explained 18.4% of the variance in health behavior engagement (R^2^ = 0.184).

### 4.3. Bootstrapping Analysis of Direct, Indirect, and Total Effects

**Figure 1:**
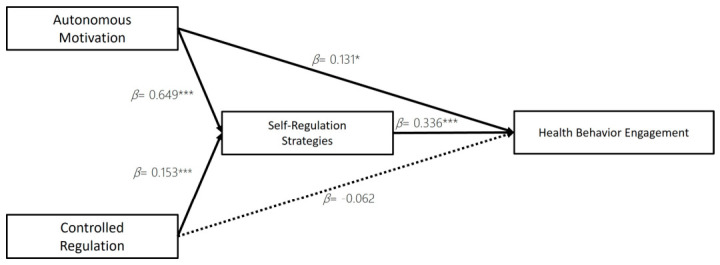
Final structural model with standardized path coefficients. Note. Solid lines indicate significant paths; the dotted line indicates a non-significant path. Values are standardized path coefficients. * *p* < 0.05, *** *p* < 0.001.

The results of the bootstrapping analysis are presented in Table 6. As indicated in Table 6, autonomous motivation had a significant positive effect on self-regulation strategies (BC 95% CI [0.648, 0.797], *p* < 0.001). In addition, both the direct effect of autonomous motivation on health behavior engagement (BC 95% CI [0.031, 0.348], *p* = 0.018) and its indirect effect through self-regulation strategies (BC 95% CI [0.206, 0.420], *p* < 0.001) were statistically significant. In contrast, controlled regulation exhibited a significant positive effect on self-regulation strategies (BC 95% CI [0.104, 0.261], *p* < 0.001), and its indirect effect on health behavior engagement was also significant (BC 95% CI [0.043, 0.131], *p* < 0.001). However, neither the direct effect (BC 95% CI [−0.216, 0.033], *p* = 0.161) nor the total effect (BC 95% CI [−0.142, 0.114], *p* = 0.777) of controlled regulation on health behavior engagement was statistically significant. Figure 1 presents the final structural model. Overall, these results indicate that self-regulation strategies partially mediate the relationship between autonomous motivation and health behavior engagement, whereas the association between controlled regulation and health behavior engagement operated primarily through an indirect pathway.

## 5. Discussion

The findings of the present study are discussed in relation to the proposed hypotheses. Specifically, the discussion below is organized around the three hypotheses concerning the associations among autonomous motivation, controlled regulation, physical activity self-regulation strategies, and health behavior engagement.

### 5.1. Hypothesis 1: Autonomous Motivation and Controlled Regulation Would Each Have Significant Effects on Self-Regulation Strategies

The results of the present study indicated that both autonomous motivation and controlled regulation had significant positive effects on self-regulation strategies. This finding suggests that the type of motivation underlying exercise participation is related not only to whether individuals engage in physical activity but also to the self-regulatory processes involved in initiating and maintaining such participation. In other words, the reasons individuals choose to exercise may be associated not only with the quantitative level of exercise behavior but also with how strategically they manage and regulate their behavior during participation. This interpretation is consistent with previous research demonstrating links between motivational and self-regulatory variables in the physical activity context, as well as with recent research identifying autonomous motivation and basic psychological need support as key explanatory factors in physical activity change [12,18].

In particular, the finding that autonomous motivation had a stronger effect on self-regulation strategies than controlled regulation indicates that individuals are more likely to actively employ self-regulatory strategies when they perceive exercise as personally important and valuable. This is consistent with recent reviews and longitudinal studies suggesting that autonomous motivation is more closely associated with physical activity participation and exercise maintenance than controlled forms of motivation [12,46,47]. Moreover, Bourque et al. [48] suggested, based on cross-sectional data, that effortful self-control may represent a psychological mechanism linking autonomous motivation to physical activity. Taken together, the present findings suggest that autonomous motivation may provide a more adaptive motivational foundation and that self-regulation strategies may be involved in the process through which such motivation is translated into actual physical activity engagement.

At the same time, the finding that controlled regulation also had a significant positive effect on self-regulation strategies suggests that controlled motivation does not function in a purely maladaptive manner. In some contexts, it may still be associated with efforts to plan and implement behavior. However, this should not be interpreted as indicating that controlled regulation represents a motivational resource of comparable quality to autonomous motivation. Previous research has demonstrated that, although controlled motivation may be associated with short-term physical activity, autonomous motivation tends to be more adaptive in the long term, and the relationship between controlled motivation and physical activity is generally less consistent [46,47,49]. From this perspective, the effect of controlled regulation observed in the present study may be understood not as a central mechanism of long-term maintenance but rather as an indicator that controlled motives may, under certain conditions, be linked to the enactment of self-regulatory efforts.

These findings imply that, to enhance self-regulation strategies, it may be more important to foster an autonomy-supportive environment that enables individuals to personally endorse the value of exercise while maintaining a sense of choice and competence, rather than emphasizing obligation or relying on normative pressure. Prior SDT-based studies and recent reviews have consistently identified autonomous motivation and need support as important correlates of health behavior change and increased physical activity [12,50,51,52]. Accordingly, interventions aimed at improving self-regulation strategies should prioritize the internalization of autonomous motivation and the provision of need-supportive feedback rather than emphasizing stronger external control.

### 5.2. Hypothesis 2: Autonomous Motivation and Controlled Regulation Would Each Have Significant Effects on Health Behavior Engagement

In the present study, autonomous motivation had a significant positive effect on health behavior engagement, whereas the direct effect of controlled regulation was not significant. According to SDT, autonomous motivation develops when individuals accept exercise as a self-endorsed and personally valued behavior, and it tends to be more stably associated with both participation in and maintenance of physical activity. By contrast, controlled regulation relies more heavily on external rewards, pressures, or the avoidance of guilt, and its association with actual behavior is therefore more limited or less consistent than that of autonomous motivation [11,53]. From this perspective, the present findings are consistent with the core propositions of SDT.

The positive effect of autonomous motivation on health behavior engagement suggests that when individuals perceive exercise as aligned with their personal interests, values, and health-related meaning, they are more likely to demonstrate higher levels of participation and sustained engagement [11,12,54]. Notably, because health behavior engagement in the present study was conceptualized as an indicator reflecting the continuity and consolidation of actual behavior rather than mere intention, it is reasonable that autonomously internalized motivation exhibited stronger explanatory power. This finding is also consistent with prior research demonstrating robust positive associations between autonomous motivation and exercise or physical activity behavior [12,13,54,55].

In contrast, the non-significant direct effect of controlled regulation on health behavior engagement suggests that motivations driven by obligation, pressure, or guilt avoidance may relate to exercise behavior to some extent but have limited capacity to explain sustained participation [56]. Recent longitudinal studies and reviews on self-determined motivation and exercise participation similarly indicate that autonomous motivation exhibits a more stable relationship with physical activity, whereas controlled motivation demonstrates less consistent patterns depending on temporal and contextual factors [12,46,47]. Furthermore, some studies suggest that external forms of controlled regulation may influence behavior indirectly through self-regulatory factors such as self-control [57], whereas autonomous motivation strengthens the pathways through which action planning and behavioral automaticity translate into actual physical activity [58]. Accordingly, controlled regulation may be better understood not as an independent motivational source that directly enhances health behavior engagement but as a supplementary factor that operates under more limited conditions [57].

These findings suggest that, to promote health behavior engagement, it is more effective to adopt an autonomy-supportive approach that enables individuals to experience exercise as a personally meaningful choice and to integrate it within their broader goals and life context, rather than emphasizing external pressure or normative expectations [12,13]. For example, supporting individuals in developing personalized exercise goals and implementation plans may be beneficial [16]. In addition, such processes are likely to be more effective within an autonomy-supportive environment characterized by opportunities for choice and non-controlling feedback [59]. Overall, these findings indicate that the enhancement and maintenance of health behavior engagement depend more fundamentally on the internalization of exercise-related values and the experience of autonomy than on external control or normative pressure [12,13].

### 5.3. Hypothesis 3: The Effects of Autonomous Motivation and Controlled Regulation on Health Behavior Engagement Would Be Mediated by Self-Regulation Strategies

The results of the present study demonstrated that self-regulation strategies had a significant positive effect on health behavior engagement and that the indirect effects of both autonomous motivation and controlled regulation were significant. These findings are consistent with recent research suggesting that exercise behavior is not determined solely by the quality of motivation but is also influenced by more proximal self-regulatory processes, such as action planning, automaticity, and self-regulatory efficacy [58,60]. In particular, autonomous motivation demonstrated both a significant direct effect on health behavior engagement and a significant indirect effect through self-regulation strategies, indicating a pattern of partial mediation. This suggests that autonomous motivation may influence behavior not only directly but also by strengthening planning- and enactment-related processes that facilitate actual participation [16,58]. In contrast, controlled regulation demonstrated no significant direct effect and only a significant indirect effect through self-regulation strategies, indicating that its influence on behavior may depend on the activation of self-regulatory processes rather than functioning as an independent and stable motivational basis, as is the case for autonomous motivation [12,47].

For autonomous motivation, these findings suggest that the more individuals perceive exercise as personally important and valuable, the more likely they are not only to demonstrate higher levels of health behavior engagement directly but also to strengthen self-regulatory processes—such as planning and preparation for action—thereby increasing actual participation [12,16]. Recent studies have reported that higher levels of autonomous motivation strengthen the pathway through which action planning develops into automatic regulation and that effortful self-control may also serve as a relevant psychological mechanism in physical activity engagement [48,58]. Accordingly, autonomous motivation appears to function as a qualitative motivational foundation for exercise behavior, whereas self-regulation strategies serve as an important enactment mechanism through which such motivation is translated into actual behavior. At the same time, caution is warranted in generalizing this interpretation to all self-regulation strategies, as previous research has primarily focused on components such as action planning, automaticity, self-regulatory efficacy, and effortful self-control rather than the full range of strategies considered in the present study [12,58,60].

In contrast, controlled regulation did not exert a direct effect on health behavior engagement but demonstrated a significant indirect effect only when mediated by self-regulation strategies. This finding suggests that pressure-based motives—such as obligation, avoidance of guilt, or meeting others’ expectations—may not independently promote sustained health behavior engagement but may be linked to behavior under conditions in which self-regulatory processes are engaged [12,47]. Recent longitudinal studies similarly indicate that controlled motivation exhibits more variable patterns than autonomous motivation, being associated with physical activity only at certain time points or even demonstrating negative associations over time, rather than maintaining a consistently positive relationship [47,57]. Some studies further suggest that external regulation may be indirectly associated with exercise behavior through self-regulatory factors such as self-control [57]. Accordingly, controlled regulation may be better understood not as an independent driver of behavior but as a supplementary factor whose influence is contingent upon the operation of proximal enactment mechanisms such as self-regulation strategies [12,57].

These findings indicate that, in improving health behavior engagement, it is important not only for individuals to possess motivation but also for that motivation to be translated into effective self-regulatory processes [16,58,60]. Accordingly, exercise-related interventions should adopt an integrated approach that simultaneously enhances autonomous motivation and supports self-regulation strategies, including concrete action planning and monitoring of implementation [12,13]. One practical approach consistent with this integrated perspective is motivational interviewing, which supports autonomous motivation by helping individuals explore ambivalence, clarify personally meaningful reasons for exercise, and develop self-endorsed goals [61]. These motivational processes can be combined with self-regulation techniques such as graded goal setting, self-monitoring, implementation planning, barrier planning, and relapse prevention, which are also consistent with behavior-change techniques identified in motivational interviewing [62]. Therefore, practitioners should help adults translate personally valued reasons for exercise into specific and manageable action plans rather than relying primarily on external pressure. In particular, for individuals with high levels of controlled motivation, it may be more effective to move beyond repeated external pressure and instead support the transformation of external demands into self-managed plans and behaviors, while gradually facilitating the internalization of exercise-related values [12,13]. Overall, the findings related to Hypothesis 3 suggest that self-regulation strategies function as an important mediating mechanism through which both autonomous motivation and controlled regulation are translated into actual exercise behavior and that interventions aimed at enhancing health behavior engagement should be designed to integrate support for both.

Several limitations should be acknowledged. First, the cross-sectional, self-report design limits causal inference and may be susceptible to common-method and social desirability biases. Second, health behavior engagement was assessed using a single-item stage-based measure, which captures readiness and participation levels but does not directly assess physical activity frequency, duration, or intensity. Third, the use of convenience sampling and online recruitment may have introduced selection bias, and adults aged 50 years and older were relatively underrepresented. Finally, the analysis involved several methodological assumptions, including the treatment of the ordinal outcome as approximately continuous.

Future research should employ longitudinal or intervention designs, incorporate objective measures of physical activity (e.g., accelerometer- or smartwatch-based indicators), and examine potential moderators such as age, gender, exercise history, fitness level, and social support. Additional mechanisms, including psychological need satisfaction, habit formation, emotional regulation, stress, and well-being, may further clarify the processes underlying sustained physical activity engagement.

## 6. Conclusions

This study examined, within the framework of SDT, whether physical activity self-regulation strategies mediate the relationships among autonomous motivation, controlled regulation, and health behavior engagement. The findings indicated that both autonomous motivation and controlled regulation were positively associated with self-regulation strategies, and that self-regulation strategies, in turn, were positively associated with health behavior engagement. However, only autonomous motivation had a significant direct effect on health behavior engagement, whereas controlled regulation was related to health behavior engagement solely through an indirect pathway via self-regulation strategies.

These findings suggest that sustained health behavior engagement depends not merely on the presence of motivation in an individual, but on the quality of that motivation and the extent to which it is translated into effective self-regulatory processes. Autonomous motivation appears to provide a more stable psychological foundation for health behavior engagement, whereas physical activity self-regulation strategies function as a key mechanism through which motivation is converted into actual behavioral enactment. Notably, autonomous motivation demonstrated both direct and indirect associations with health behavior engagement, whereas controlled regulation appeared to influence behavior only when accompanied by self-regulatory processes.

Overall, the present study highlights the importance of integrating motivational quality and self-regulation in explaining health behavior engagement. From a practical perspective, interventions aimed at enhancing adults’ engagement in health behaviors should move beyond simply emphasizing the necessity of exercise. More effective approaches are likely to involve supporting individuals in internalizing the value and meaning of exercise, while also facilitating the development of concrete self-regulation strategies, such as goal setting, self-monitoring, time management, and implementation monitoring. Ultimately, sustained health behavior engagement may be best promoted by fostering both autonomy-supportive environments and the self-regulatory resources needed to maintain action.

## Figures and Tables

**Table 1:** Background Characteristics of the Participants (*n* = 468).

Variable	Category	*n*	%
Gender	Male	227	48.5
	Female	241	51.5
Age	20 s	134	28.6
	30 s	119	25.4
	40 s	137	29.3
	50 and older	78	16.7
Exercise intensity	Low	157	33.5
	Moderate	167	35.7
	High	144	30.8

**Table 2:** Results of Confirmatory Factor Analysis for the Self-Determination and Self-Regulation Scales.

Variable		β	B	SE	C.R.	CR	AVE	α
External regulation	External regulation1	0.867	1.000			0.799	0.581	0.748
	External regulation2	0.851	1.001	0.056	17.768			
	External regulation3	0.518	0.883	0.080	11.070			
Introjected regulation	Introjected regulation1	0.820	1.000			0.821	0.606	0.819
	Introjected regulation2	0.789	0.999	0.063	15.732			
	Introjected regulation3	0.723	0.955	0.064	14.866			
Identified regulation	Identified regulation1	0.804	1.000			0.826	0.613	0.825
	Identified regulation2	0.753	0.988	0.063	15.673			
	Identified regulation3	0.790	1.053	0.065	16.241			
Integrated regulation	Integrated regulation1	0.889	1.000			0.879	0.709	0.825
	Integrated regulation2	0.794	0.752	0.036	20.939			
	Integrated regulation3	0.840	1.008	0.044	22.808			
Intrinsic motivation	Intrinsic motivation1	0.935	1.000			0.937	0.832	0.935
	Intrinsic motivation2	0.896	1.053	0.033	31.846			
	Intrinsic motivation3	0.905	0.989	0.030	32.608			
χ^2^ = 224.485, df = 80, *p* < 0.001, CMIN/df = 2.806, IFI = 0.965, TLI = 0.954, CFI = 0.965, RMSEA = 0.062								
Goal setting	Goal setting1	0.849	1.000	–	–	0.847	0.735	0.847
	Goal setting2	0.865	1.000	0.050	20.07			
Self-monitoring	Self-monitoring1	0.747	1.000	–	–	0.848	0.739	0.833
	Self-monitoring2	0.959	1.196	0.072	16.659			
Time management	Time management1	0.851	1.000	–	–	0.834	0.716	0.833
	Time management2	0.841	1.073	0.052	20.45			
Eliciting social support	Eliciting social support1	0.791	1.000	–	–	0.805	0.674	0.804
	Eliciting social support2	0.85	1.056	0.085	12.451			
Relapse prevention	Relapse prevention1	0.84	1.000	–	–	0.883	0.792	0.880
	Relapse prevention2	0.937	1.077	0.052	20.559			
Reinforcement	Reinforcement1	0.804	1.000	–	–	0.807	0.676	0.805
	Reinforcement2	0.840	0.980	0.058	16.756			
χ^2^ = 109.18, df = 39, *p* < 0.001, CMIN/df = 2.799, IFI = 0.979, TLI = 0.964, CFI = 0.979, RMSEA = 0.062								

Note. β, standardized coefficient; B, unstandardized coefficient; SE, standard error; C.R., critical ratio; CR, composite reliability; AVE, average variance extracted; α, Cronbach’s alpha; CMIN/df, minimum discrepancy divided by degrees of freedom; IFI, incremental fit index; TLI, Tucker–Lewis index; CFI, comparative fit index; RMSEA, root mean square error of approximation.

**Table 3:** Composition of the Parsimonious Structural Model.

Construct	Indicators	Measurement
Controlled regulation	External regulation, introjected regulation	Mean of subfactor scores
Autonomous motivation	Identified regulation, integrated regulation, intrinsic motivation	Mean of subfactor scores
Self-regulation strategies	Goal setting, self-monitoring, time management, eliciting social support, reinforcement, relapse prevention	Mean of subfactor scores
Health behavior engagement	Single stage-of-change item	Recoded single-item score (1–5)

Note. Controlled regulation, autonomous motivation, and self-regulation strategies were modeled as latent constructs using subfactor scores as indicators. Health behavior engagement was entered as a single observed outcome variable based on the recoded stage-of-change item.

**Table 4:** Means, Standard Deviations, Pearson’s Correlations, Skewness, and Kurtosis of the Study Variables.

Variables	a	b	c	d	e	f	g	h	i	j	k	l
a. external regulation	1.000											
b. introjected regulation	0.134 **	1.000										
c. identified regulation	−0.347 **	0.137 **	1.000									
d. integrated regulation	−0.315 **	0.311 **	0.464 **	1.000								
e. intrinsic motivation	−0.373 **	0.087	0.315 **	0.628 **	1.000							
f. goal setting	−0.182 **	0.251 **	0.318 **	0.495 **	0.352 **	1.000						
g. self-monitoring	−0.024	0.250 **	0.159 **	0.405 **	0.282 **	0.547 **	1.000					
h. time management	−0.209 **	0.233 **	0.312 **	0.541 **	0.464 **	0.692 **	0.609 **	1.000				
i. eliciting social support	−0.073	0.109 *	0.172 **	0.413 **	0.435 **	0.250 **	0.288 **	0.342 **	1.000			
j. relapse prevention	−0.216 **	0.298 **	0.416 **	0.514 **	0.405 **	0.508 **	0.483 **	0.521 **	0.405 **	1.000		
k. reinforcement	−0.206 **	0.180 **	0.292 **	0.481 **	0.424 **	0.445 **	0.478 **	0.508 **	0.493 **	0.566 **	1.000	
l. health behavior engagement	−0.214 **	0.117 *	0.157 **	0.312 **	0.342 **	0.409 **	0.272 **	0.468 **	0.169 **	0.256 **	0.274 **	1.000
Mean	1.729	2.538	4.308	3.515	3.654	3.377	2.844	3.270	3.067	3.574	3.341	4.216
Standard deviation	0.857	1.012	0.678	0.966	1.078	1.049	1.177	1.115	1.149	0.978	1.064	1.060
Skewness	1.093	0.147	−0.951	−0.211	−0.572	−0.454	0.068	−0.275	−0.132	−0.658	−0.353	−1.110
Kurtosis	0.553	−0.694	0.937	−0.614	−0.383	−0.386	−0.884	−0.626	−0.846	0.092	−0.505	0.223

Note. Values on the diagonal are 1.00. All correlation coefficients are Pearson’s product-moment correlations. Skewness and kurtosis were examined to assess univariate normality. * *p* < 0.05, ** *p* < 0.01.

**Table 5:** Structural Path Estimates of the Saturated Model.

Independent Variable	Dependent Variable	β	B	SE	C.R.	*p*-Value	R^2^
Controlled Regulation	Self-Regulation Strategies	0.153	0.179	0.040	4.445	<0.001	0.445
Autonomous Motivation	Self-Regulation Strategies	0.649	0.723	0.038	18.835	<0.001	-
Controlled Regulation	Health Behavior Engagement	−0.062	−0.094	0.064	−1.462	0.144	0.184
Autonomous Motivation	Health Behavior Engagement	0.131	0.187	0.079	2.358	0.018	-
Self-Regulation Strategies	Health Behavior Engagement	0.336	0.432	0.072	5.981	<0.001	-

Note. β, standardized coefficient; B, unstandardized coefficient; SE, standard error; C.R., critical ratio; R^2^, squared multiple correlation. R^2^ values indicate the proportion of variance explained in each endogenous variable. The final structural model was specified as a saturated model; therefore, global fit indices were not interpreted.

**Table 6:** Bootstrap Results for Direct, Indirect, and Total Effects in the Saturated Model.

Path	Effect Type	BC 95% CI Lower	BC 95% CI Upper	*p*-Value	Result
Autonomous Motivation → Self-Regulation Strategies	Direct	0.648	0.797	<0.001	Significant
Controlled Regulation → Self-Regulation Strategies	Direct	0.104	0.261	<0.001	Significant
Self-Regulation Strategies → Health Behavior Engagement	Direct	0.285	0.570	<0.001	Significant
Autonomous Motivation → Health Behavior Engagement	Direct	0.031	0.348	0.018	Significant
Controlled Regulation → Health Behavior Engagement	Direct	−0.216	0.033	0.161	Not significant
Autonomous Motivation → Self-Regulation Strategies → Health Behavior Engagement	Indirect	0.206	0.420	<0.001	Significant
Controlled Regulation → Self-Regulation Strategies → Health Behavior Engagement	Indirect	0.043	0.131	<0.001	Significant
Autonomous Motivation → Health Behavior Engagement	Total	0.371	0.619	<0.001	Significant
Controlled Regulation → Health Behavior Engagement	Total	−0.142	0.114	0.777	Not significant

Note. BC 95% CI, bias-corrected 95% confidence interval. Indirect effects were considered significant when the bias-corrected 95% confidence interval did not include zero. Health Behavior Engagement was entered as the observed outcome variable in the final parsimonious structural model.

## Data Availability

The data presented in this study are not publicly available due to ethical restrictions and the inclusion of sensitive participant information, but are available from the corresponding author upon reasonable request.

## Abbreviations

The following abbreviations are used in this manuscript:

β	standardized coefficient
B	unstandardized coefficient
SE	standard error
C.R.	critical ratio
CR	composite reliability
AVE	average variance extracted
α	Cronbach’s alpha
BC 95% CI	bias-corrected 95% confidence interval
χ^2^	chi-square
df	degrees of freedom
CMIN/df	minimum discrepancy divided by degrees of freedom
IFI	incremental fit index
TLI	Tucker–Lewis index
CFI	comparative fit index
RMSEA	root mean square error of approximation
